# Event and Comment

**Published:** 1922-02

**Authors:** 


### DENTAL REGISTER

THE MAGAZINE OF DENTISTRY

Vol. LXXVI FEBRUARY, 1922 No. 2

### EVENT AND COMMENT

**Our Filling Materials and Methods.** Dr. James M. Prime, of Omaha, has been making some investigations as to the materials and methods used by about thirty operators, and he reports some of his conclusions in an article published in the *Journal of the National Dental Association.* His deductions are somewhat unsatisfactory, as such findings can not be expected to give accurate results when examined by scientists when dealing with clinical problems of more or less practical subjects. His conclusions as to the comparative values of principles of practice as ordinarily studied necessarily show differences as to both materials and methods; as usual, the artistic is always less exact than these scientific findings will show. He tabulated 28,000 operations in the use of the four principal materials: malleted gold foil, gold inlays, amalgam, and silicious cements. The several materials were used in the following proportions: malleted gold foil, 26%; cast gold inlay, 29%; amalgam, 34%; silicate cement, 11%. Undoubtedly a larger number of operators, in a given section of the country, because of the place in which they secured their technical instruction, would give various reactions to the same materials because of this variety of teaching. We doubt if any such tabulation would reveal a true conception of actual results in the same schools, to say nothing of the entire country.

Nevertheless, this tabulation is valuable and it tells us something as to the preferences of some groups of practitioners and their known methods of manipulating material. For lack of a fixed standard of procedure and materials used, this research may set a starting place from which we can at least accumulate data which by experimental use we should be able to improve our technic or even change both it and the materials we use.

Obviously, it would be unwise for us to dogmatize on this topic without better knowledge of the physical characteristics of the materials we employ, or with the methods adopted in using them. As both are subject to constant changes, we should wait until our science is more definitely assured and our technic more accurately demonstrated. We can scarcely hope to reach accurate results and uniformity in methôds that will be generally adopted in practice until more complete and exhaustive researches confirm our various ideas and methods of practice.

Marshall H. Webb probably personified our highest attainments as a malleted gold foil operator; and G. W. Black attained more than any other operator—the standard of cavity preparation for this class of filling; and many others have made important contributions to the present ideals and achievements in this department, but as yet no ideal filling material or method of operating is generally approved, and certainly none has as yet been so adopted.

We have in a word not as yet found an ideal material nor discovered the best way to prevent the destruction of the natural tooth by caries or accidents. To make a statement like this, in view of all that we have done to save the teeth from hopeless loss, may seem unwarranted, but we are forced to recognize the fact that we have now no system of repair or therapy that has seriously checked dental caries of the teeth and absolute loss sooner or later. Do we know at the present time how to fill teeth so as to keep them safe and capable to the end of our lives? We have learned many things of incalculable value from our scientific and technical arts, but we are not yet in a position to pass final judgment on so complicated a technical proposition. This is not because it is so intricate a method, but because its biological environments must be considered with more intimate correlation for adequate knowledge to obtain even technical excellence.

We have made wonderful progress in technical art, and we are developing new means of preserving and utilizing the normal functions of the body. We still have much to learn of nature's vital processes, and we sometimes wonder if it will ever be practicable to successfully cope with the ever increasing pathologic environment under which we live and struggle. We no sooner discover and master some part of our technical art program than we find it undermined by some insiduous microbe to counterbalance our most beautiful and artistic devices, which destroys their usefulness and many times causes them to become a serious menace to health and happiness.

Today we seem to have laid down the fight for the time being, at least, so that our prosthetic activities shall have a chance to resuscitate the lame and crippled denture with crown and bridge restorations; or until the slaughter of the innocents shall have ceased from troubling by the "*focalists*" and exodontists, so as to get a breathing spell that we may, if it is practicable, contrive suitable methods of prosthesis with which to carry on some proper substitutes for the normal functions of mastication and insalivation.

Life indeed seems hard, but we shall need to work harder to increase our vigil of watching and striving to overcome all handicaps of the evil forces that we may ultimately have the knowledge and happiness of overcoming perverse nature and inaugurating a better era of health.

In some ways we seemingly have suffered defeat, but in the meanwhile we have enjoyed the struggle and still believe we shall see the coming of a better day. Is it not already a good promise to know that more today are studying the whole health problem scientifically than ever before? We may need to retrace our steps and study anew the problems of infant nutrition, as well as children's hygiene, orthodontia, prophylaxis, dental caries, and operative dentistry, before we have to further contend with dental surgery and prosthodontia.

